# Medical Opinion and Movement

**Published:** 1909-06-12

**Authors:** 


### Medical Opinion and Movement.

PROFESSOR BERKLEY, of Baltimore, publishes in the *Johns Hopkins Hospital Bulletin* the results of some researches into Acute Alcoholic Poisoning and its effects upon nervous and vascular tissues. The histological studies have been chiefly upon rabbits, but some of the effects of alcohol ingestion on vascular function were observed in human beings who volunteered to be made the subjects of experiment. It is concluded that in acute alcoholic poisoning the stress of the action of the drug falls upon the walls of the blood-vessels rather than upon the nervous elements of the brain. The involvement of the latter is more gradual than that of the mesoblastic tissues, and is only traceable after the lymphatic channels have become blocked with the detritus of white corpuscles and other cellular elements. Nevertheless the action of ethyl alcohol, in more moderate but more prolonged dosage, is to deteriorate the nerve cells, as evidenced by nuclear and dendritic changes. In its action on nervous tissues alcohol resembles strongly the toxalbumins and ricin, and its effect is proportionate to the quantity administered, and to the duration of its poisonous action before death ensues.

IN the *Munchener Medicinische Wöchenschrift*, Lichtenberg and Muller deal with the question of Post-operative Thoracic Conditions. The authors have examined more than 100 successive cases, in which extensive abdominal operations have been performed, and have come to the conclusion that lung complications are extremely common after operations implicating the abdominal viscera. Slight rises of temperature of transient duration following the operation are almost invariably due to slight engorgement of the lungs. The prognosis in such cases is good; the patients recover rapidly, and unless carefully sought for, the thoracic complication is probably overlooked. They usually arise on the second, third, or fourth day after the operation, and the authors consider that this frequency has nothing whatever to do with the prevalence of general anæsthesia. Their importance lies in the fact that they may in rare cases develop into typical post-operative pneumonias. The authors consider that such pneumonias directly due to chloroform or ether narcosis are exceedingly rare. With regard to heart complications the authors conclude that these play a relatively slight *rôle* after abdominal operations. Where an organic cardiac lesion already exists the condition of the myocardium may be altered by the operation, and there may be changes of some importance in the vascular system as tested by taking the blood-pressure. In general the practitioner's attention must be concentrated upon the pulmonary condition, and only in rare cases will the cardiac lesions call for treatment. Out of their 100 cases, no less than 73 showed signs of consolidation, engorgement, or bronchitis, following the operation after an interval which varied from two to six days, but in no case was a death attributable to these conditions.

A RECENT number of the *Zentralblatt für Chirurgerie* contains an account of some experiments performed by MacLennan with a view to the determination of the Functions of the Thymus Gland. The author took a litter of rabbits, aged six weeks, and removed the organ from each animal. He found that the operation made the animals more vigorous and increased their resistance to various infections, such as tuberculosis, favus, enteritis, etc. Moreover, the removal of the gland had a marked influence on the diminution in volume of the thyroid body. The author therefore thinks that the thymus should be examined in all persons suffering from Graves' disease with a view to its extirpation if it should be found enlarged.

DANIELOPOLU pleads for the greater employment of Strophanthine in Cardiac Lesions. In his opinion it is one of the most valuable tonics at the disposal of the practitioner. It is rapid in action, acts, according to him, exclusively on the heart, and its dosage can be graduated to a nicety in each individual case. The author employs the intravenous injection method, as his experience has led him to the conclusion that subcutaneous injections are more painful and give rise to local irritation. He uses a miligram of the drug, and repeats the dose when necessary twenty-four hours later. In cases accompanied by much œdema, he has found the most marked benefit. The amount of urine excreted is rapidly increased, the œdema is improved, and the general condition much ameliorated. Nevertheless the diuretic action is often insufficient, especially in cases of old standing heart lesions, when the kidneys are affected; in such cases the author uses theobromine, or theocine in combination with strophanthus.

JACQUET and Jourdanet, in *La Rèvue de Médecine*, discuss the ætiology and therapeutics of Migraine. In the authors' opinion, migraine is most certainly influenced by the condition of the stomach, and can be favourably affected by careful attention to the diet. They cite a series of cases in which treatment consisted merely in a strict dietary. Tea, coffee, alcohol, and liqueurs are forbidden, and all condiments except salt. Fish, shellfish, and all pork-butcher's meat except lean ham are forbidden. Prolonged and careful mastication of all food, even the most tender, is insisted on as essential to success, for victims of migraine often indulge to excesses in food, drink, and condiments; but, above all, they are rapid eaters. Prolonged mastication would seem to have two effects: lessening the work of the stomach by excluding indigestible material from the diet, and reducing gastric irritability to a minimum. The drawback to the treatment lies in the difficulty experienced in securing obedience from the patient. The authors, however, produced great benefit in almost every one of their series of sixteen cases so treated.

THE Subcutaneous Injection of Sea Water is being taken up by one or two enthusiasts in America as the latest cure for all and sundry diseases. The idea seems to have been started by Robert-Simon in Philadelphia last winter, and now Dr. Le Boutillier has read a paper on the subject before the Pædiatric Society of Pennsylvania. The treatment, he says, follows rationally the theory of the marine origin of life, and the fact that blood serum and sea water contain the same chemical constituents in approximately the same proportions. Now wherever life originated the date is immeasurably remote, and it is quite safe to assert that at that time the composition of the sea was very different indeed from what it is now, for the density of the sea is known to be slowly increasing. To regard the blood serum and sea water as fluids of approximately the same composition seems equally fantastic: even were it so, it would have to be established first that blood serum will cure every ailment before the argument can be extended to sea water. The cases narrated in support of Dr. Le Boutillier's treatment were adversely criticised by every subsequent speaker, and the whole society seemed unwilling to take the author seriously; but if his paper should fall into the hands of a certain type of newspaper sensationalist in this country, we may nevertheless soon expect to be asked by inquisitive members of the lay public for details of this latest cure for some pestilence or other from which they or their friends are suffering.

---

THE Excision of Thrombosed Veins in Puerperal Pyæmia is advocated in the *American Journal of Obstetrics* by Professor Whitridge Williams, who reports five cases with four complete successes. As a result of collating several large published series of cases of puerperal pyæmia accompanied by thrombosis, it is concluded that the mortality of this condition under expectant treatment is not less than 60 per cent. The operation of ligature of the veins by which the infection spreads from the uterus seems to have been suggested by several obstetricians, but the first who took it up and performed it were Trendelenburg and Bumm, independently, who published their results in 1902. Professor Williams has searched the literature very thoroughly to date, and has collected in all 56 cases. Of these fifteen were done by the extra-peritoneal route, but the author has several objections to urge against this, and Trendelenburg has abandoned it in favour of the trans-peritoneal. Peritonitis, which it was feared might follow the latter operation, has only been found to do so twice in 41 cases, whereas it developed once after the extra-peritoneal incision. As regards indications for the operation, the author would lay it down as necessary whenever a positive diagnosis can be made; when thrombosed vessels cannot be palpated *per vaginam* the abdomen should be opened if the patient is seriously ill and the clinical symptoms show no signs of improvement, provided that peritonitis or abscess in the broad ligament has not developed.

THE frequency of Pulmonary Tuberculosis in Children, or rather of phthisis of the adult type, is discussed by Dr. Sawyer in the *British Journal of Children's Diseases.* It appears from the returns of the medical inspectors of schools that great divergencies exist in different places in the incidence of chronic pulmonary tuberculosis amongst children, or else that the interpretation of various physical signs is not by any means uniform. Thus one (lady) inspector discovered and reported 15.5 per cent. of cases among 1,507 school children, a proportion so enormous that the accuracy of the diagnoses may not unreasonably be impeached out of hand. Other medical officers report percentages varying from 6 down to 0.1. Dr. Sawyer approaches the question from a slightly different aspect, for his figures are based not upon the examination of a large number of nominally healthy children, but on an analysis of his outpatient cases at the General and Children's Hospitals, Birmingham; and, as he points out, it is hardly likely that the percentage of phthisis present is less than that of the general non-adult population. Among 8,000 children examined, phthisis of the adult type was diagnosed in fifteen only; four of these recovered so completely that the diagnosis is presumed to be incorrect, and one other has also improved, has no tubercle bacilli in the sputum, and is now regarded as probably suffering from bronchiectasis. Of 11 others marked doubtful after the first examination nearly all improved under treatment, and were ultimately lost sight of. There were also cases of acute miliary and broncho-pneumonic tuberculosis, which are not included in these figures, and there were two instances of tuberculous peritonitis in which the lungs also were demonstrably affected. But this does not affect the point that only 10 children out of 8,000 were found to be the subjects of chronic or latent phthisis of adult type, and nearly all of them were near the age limits of the hospitals, that is just under or about puberty. The same infrequency of this lesion is brought out by the tables of autopsies on children at the General Hospital. Out of 22 children dying of tuberculous disease of the lungs, only two presented lesions of adult type, the remaining 20 being all of acute and miliary form.

---

A SIMPLE method of operation for the cure of Prolapse of the Rectum in children has been devised by Dr. G. Ekehorn, of Sundsvall. The prolapse is reduced, and the surgeon introduces the left index finger into the rectum. A curved needle is driven into the skin along the lower part of the free border of the sacrum, and is carried through all the soft parts to the interior of the rectum, where it meets the index finger. A thick silk suture is then threaded through the point of the needle, and the needle is withdrawn. The same proceeding is carried out on the opposite side, and the other end of the same suture drawn through. The two ends of the suture are then tied across on the skin in the sacral region. The suture is left in position for a couple of weeks and then withdrawn. Dr. Ekehorn has used this method of treatment four times with complete success. The first patient was seven years old, and the last three months. In each case the operation was effective, and there was no return of the prolapse. The operation is certainly simple, and the author claims that it is rational, as it holds the rectal wall against the sacrum, where it should naturally take its support.

Moszkowicz, of Vienna, first conceived the idea of inducing hyperæmia by a constricting band round the limb as a means of testing the condition of the blood-vessels and determining the site for Amputation in cases of spontaneous Gangrene of the Foot. The method has also been adopted by Mendelsohn of Strassbourg, by Bergemann of Königsberg, and by Lejars of Paris, and the latter contributes an interesting article on the subject to *La Semaine Médicale*. The test is carried out in the following manner: The two limbs are elevated for some minutes, and an indiarubber bandage is bound round each thigh high up, sufficiently tight to produce complete hæmostasis in the limbs. The bandages are left in position for five minutes, and then withdrawn and the results observed. On the healthy side the red coloration suffuses down the limb in a few moments as far as the toes. On the gangrenous side it may also extend as far as the foot to the limits of the gangrene, but more often it ceases at a variable height in the leg, or it may not pass beyond the knee. In this way an indication is given as to the level at which amputation may with safety be carried out. Usually on the diseased side the hyperæmic coloration appears more slowly, and does not end with a clear line of demarcation, but has an indefinite limitation which is often lower on one aspect of the limb than on another. These points must all be taken into consideration in "reading" the indications for amputation and the kind of flaps best adapted to the case. Several cases are reported in which this test has been of considerable service and in which amputation has been carried out with satisfactory results at a lower level than appeared to be justified by other indications, such as the pulsation of the arteries. It might be suggested that the hyperæmic congestion induced in such cases is in itself not without danger, but, according to these authors, no untoward accident or inconvenience has followed the use of this hyperæmic test.

At a meeting of the Berlin Society of Medicine Dr. C. Hamburger read an interesting paper on the use of fluorescine in the study of the Physiology and Pathology of the Human Eye. Ehrlich of Frankfort has shown that the sodium compound of fluorescine (uranin) has such a colouring power that it is possible to detect its presence on a dark background in solution of 0.000004 strength by reason of the green fluorescence that it gives to the solution. It has been used to show that the anterior chamber of the eye is completely separated from the posterior chamber. In spite of the extreme diffusibility of fluorescine, its injection into the posterior chamber in animals is unable to overcome the barrier separating this from the anterior chamber in a healthy eye; whereas, if there is a lesion, the aqueous humor becomes tinted, and the rapidity and intensity of this coloration depend upon the severity of the morbid condition. Ehrlich foresaw the utility of this test in human pathology, but the supposition that uranin is highly toxic has hitherto prevented its use. Dr. Hamburger, however, is able to give the assurance that this fear of toxicity is entirely unfounded. He has injected uranin in infants and in the aged in doses of 6 to 8 grammes without any ill effects, and he advocates its use as a means of diagnosis in inflammatory conditions of the iris and ciliary body, and especially in glaucoma. In such cases the fluorescine penetrates the barrier to the anterior chamber and the eye becomes coloured, as the rest of the body after a subcutaneous injection, but when the inflammation involves the superficial tissues only (conjunctivitis or superficial traumata) there is no coloration of the eye.

The much-vexed question of the Ætiology of Rickets is discussed by Dr. Aron in the *Biochemische Zeitschrift* from the point of view of insufficiency of calcium salts in the food. The researches of this author have led him to form the opinion that the supply of calcium salts plays an important *rôle* in the ætiology of this disease, and that in a large number of cases, especially in breast-fed infants, the quantity ingested is insufficient. By giving young and growing animals a food poor in lime salts Dr. Aron has been able to produce bony lesions which in all respects resembled those found in the rachitic skeleton—deformities of the bones, swellings of the lower ends of the radius and femur, proliferation of the cartilaginous and periosteal zones. Chemical analysis confirmed the morphological data: the bones of these animals contain a larger proportion of water and less dry residue, and the dry residue contains less lime salts than in normal bones. There is also a further resemblance in the lime content of the other organs. In the rachitic condition and also in these animals the author finds that the other tissues of the body contain a normal proportion of lime salts. By computing the average amount of lime salts contained in human milk and the amount usually required by a growing infant, the author estimates that there is only just sufficient, and there is no margin for cases in which the milk may be poor in lime or the infant's digestive organs unable to absorb the full amount ingested. Moreover, the author finds that insufficiency of lime salts is often accompanied by an abundant supply of milk, which tends to increase the disproportion between the growth of the child and the supply of lime salts. For a child growing rapidly an extra supply of calcium salts is especially needed, and should be added in the form of tricalcium phosphate 1 gramme per day. The fact that the administration of lime salts does not appear to affect the disease, once established, does not, in the opinion of the author, militate against this theory. The same phenomenon was observed in the case of the animals experimented upon. When the lesions were established the addition of lime salts to the food only affected the condition very slowly, often after several months.

